# Reversible severe pulmonary hypertension related to scurvy in children

**DOI:** 10.1186/s12872-023-03629-6

**Published:** 2024-01-03

**Authors:** Marin Satawiriya, Apichai Khongphatthanayothin, Alisa Limsuwan

**Affiliations:** 1 Division of Pediatric Cardiology, Department of Pediatrics, Faculty of Medicine Ramathibodi Hospital, Mahidol University, 270 Rama 6 Rd, Rachathewi, Bangkok, 10400 Thailand; 2 Bangkok Heart Hospital, Bangkok, Thailand; 3 Center of Excellence in Arrhythmia Research Chulalongkorn University, Department of Medicine, Faculty of Medicine, Chulalongkorn University, Bangkok, Thailand

**Keywords:** Pulmonary hypertension, Scurvy, Pulmonary hypertensive crisis, Children

## Abstract

**Background:**

Severe pulmonary hypertension (PH) in childhood is rare and can manifest as a life-threatening episode. We present 2 children with restrictive dietary habits with severe pulmonary hypertension secondary to scurvy and iron deficiency anemia with treatment and outcome.

**Case presentation:**

The first case is a 2-year-old boy who presented with vomiting, diarrhea, and fever. After rehydration, he had recurrent episodes of hypotension with intermittent abdominal pain. Fluid resuscitation and inotropic medication were given. Then he suddenly collapsed. After 4-min cardiopulmonary resuscitation, his hemodynamic was stabilized. Most of the medical workup was unremarkable except for PH from the echocardiogram with estimated systolic pulmonary artery pressure (PAP) at 67 mmHg. Transient PH was diagnosed, and milrinone was prescribed. Since he had restrictive dietary habits and sclerotic rim at epiphysis in chest films, his vitamin C level was tested and reported low-level result. The second case is a 6-year-old boy with acute dyspnea, a month of low-grade fever, mild cyanosis, and a swollen left knee. Echocardiogram indicated moderate TR with estimated systolic PAP at 56 mmHg (systolic blood pressure 90 mmHg). Milrinone was given. Right cardiac catheterization showed PAP 66/38 (mean 50) mmHg and PVRi 5.7 WU.m^2^. Other medical conditions causing PH were excluded. With a history of improper dietary intake and clinical suspicion of scurvy, vitamin C was tested and reported undetectable level. Administration of vitamin C in both cases rapidly reversed pulmonary hypertension.

**Conclusion:**

Pediatric PH related to vitamin C deficiency can manifest with a wide range of symptoms, varying from mild and nonspecific to severe life-threatening episodes characterized by pulmonary hypertensive crises. PH associated with scurvy is entirely reversible with appropriate investigation, diagnosis, and treatment. Our report highlights the importance of considering nutritional deficiencies as potential confounding factors in pediatric PH, emphasizing the need for comprehensive evaluation and management of these patients.

## Introduction

Severe pulmonary hypertension in childhood is considered a rare condition with dismal outcomes [1]. Initial presentation typically has nonspecific symptoms. PH can first manifest as a life-threatening episode. We described 2 children who presented with elevated pulmonary artery pressure (PAP) and decreased cardiac output (CO), leading to pulmonary hypertensive crises (PHC) in one case. Investigation of the underlying cause and disease progression revealed reversible PH secondary to scurvy.

## Case 1

**Fig. 1:**
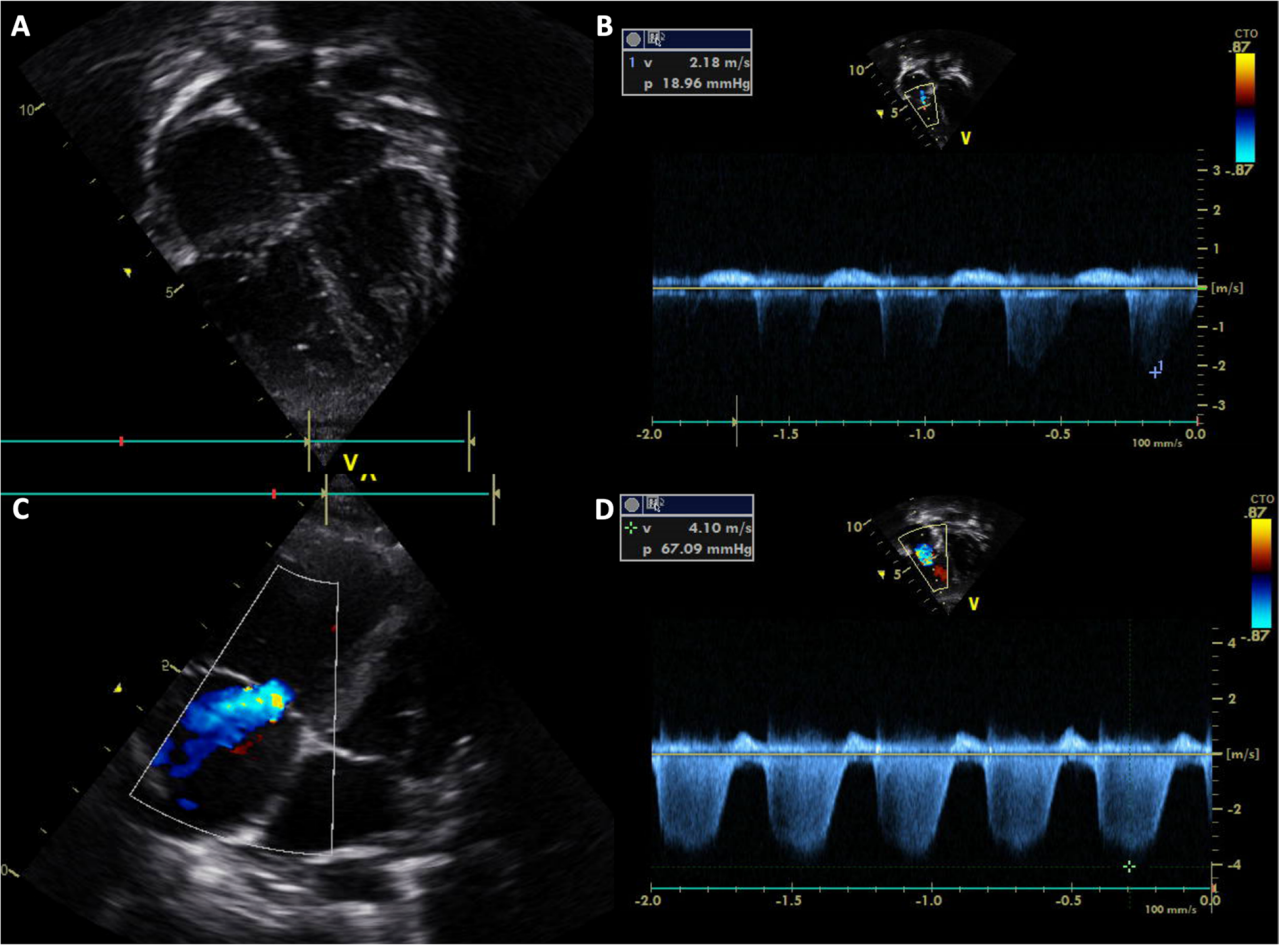
Echocardiographic illustration of Case 1 The initial 2D echocardiogram showed normal cardiac structure (**A**) with a tricuspid regurgitation (TR) velocity of 2.18 m/s (**B**). After cardiac arrest, the colored echocardiogram demonstrated moderate TR (**C**) with peak velocity of 4.1 m/s (**D**)

**Fig. 2:**
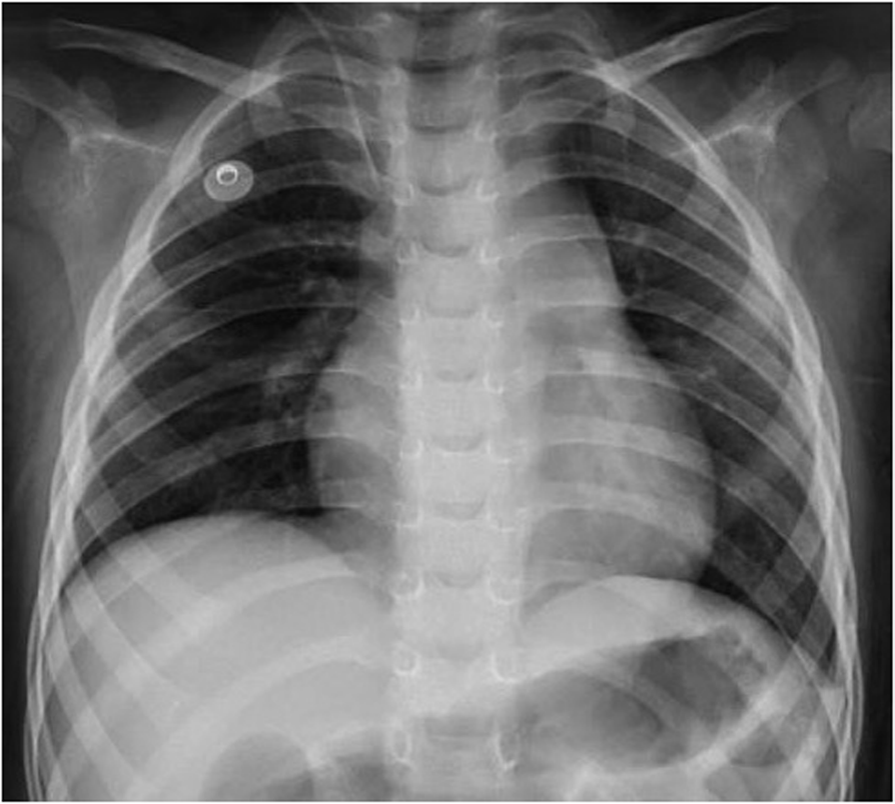
Chest film revealed mild cardiomegaly and decreased pulmonary blood flow

**Fig. 3:**
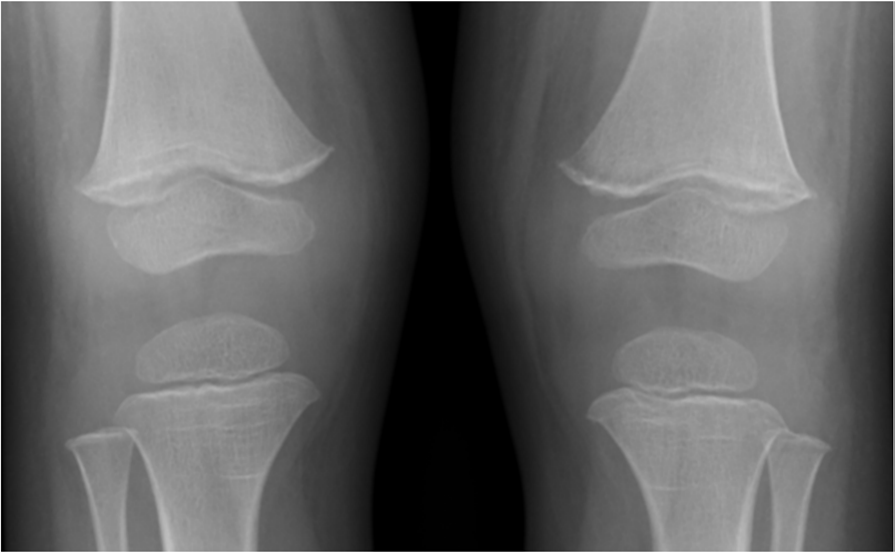
Both knees x-ray showed sclerotic rim at epiphysis

A 2-year-old boy presented with vomiting, diarrhea, and fever for 5 days. During the hospitalization for rehydration, he had couple episodes of hypotension along with abdominal cramping. Initial echocardiographic findings were unremarkable with normal estimate PAP (Fig. 1A and B). He was given intravenous fluid resuscitation and then was transferred to the pediatric intensive care unit where he suddenly collapsed and had confirmed cardiac arrest. After 4-min cardiopulmonary resuscitation, his hemodynamic was restored and maintained with inotropic medications. The initial investigation was unremarkable. Immediately post-resuscitation, the echocardiographic findings revealed dilated right atrium (RA) and right ventricle (RV) with moderate tricuspid regurgitation (TR) with peak TR velocity of 4.1 m/s (Fig. 1C and D). The estimated systolic RV pressure was 67 mmHg (Fig. 1D). Diagnosis of PH was made and further investigations for PH were initiated, including pulmonary computer tomographic angiography (CTA). The patient was kept on a ventilator with high oxygen concentration and milrinone was started before the patient was transferred to our pediatric PH center. At our institute, an initial physical examination revealed a loud S2 without significant heart murmur. Chest film revealed mild cardiomegaly, prominent central pulmonary artery, and decreased pulmonary vasculature (Fig. 2). Echocardiographic findings illustrated RA and RV enlargement, and mild TR with peak TR velocity of 3 m/s and an estimated systolic PAP of 29 mmHg. Three days after referral, the right cardiac catheterization showed the PAP of 28/13, mean = 20 mmHg. The derived calculated pulmonary vascular resistance index (PVRi) was 1.8 WU.m^2^. All available viral and genetic studies were negative. From the retrospective interview, his development was mildly delayed and he also had restrictive eating patterns. His dietary preference were rice, pork, eggs, and milk, with very few vegetables and fruits. Further investigation for nutritional deficiency was explored. The radiographic finding revealed the sclerotic rim at epiphyses in both femurs (Fig. 3). The vitamin C level was undetectable. Daily 300 mg of vitamin C was given. He also had iron deficiency anemia which he was treated with iron supplement. After he was discharged from the hospital, his symptoms gradually improved and returned to his usual state of health within 2 weeks. One-year follow-up, he has been well with normal PAP.

## Case 2

**Fig. 4:**
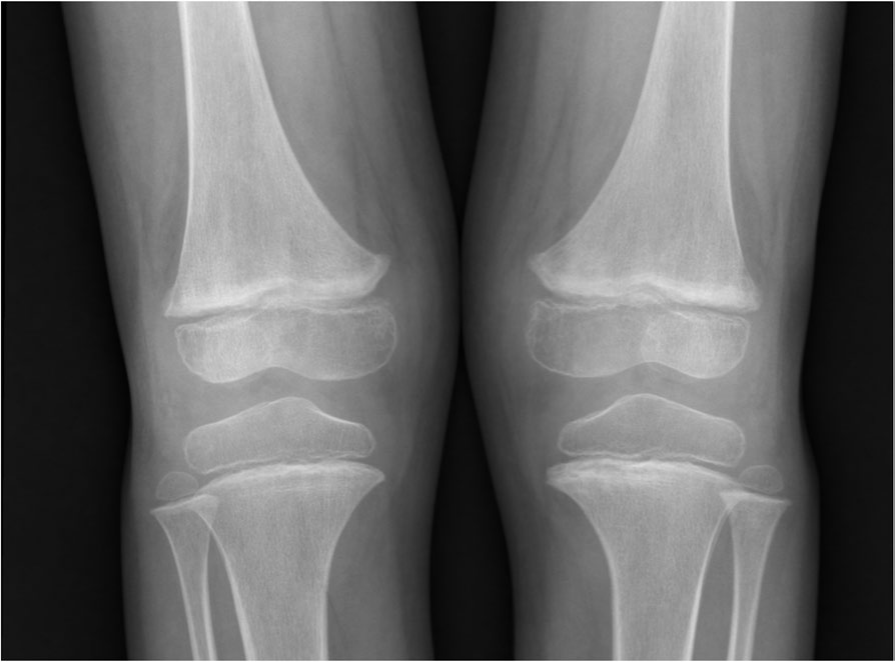
Both knees x-ray showed metaphyseal fraying and hypolucent lesions at metaphyses of both femurs

**Fig. 5:**
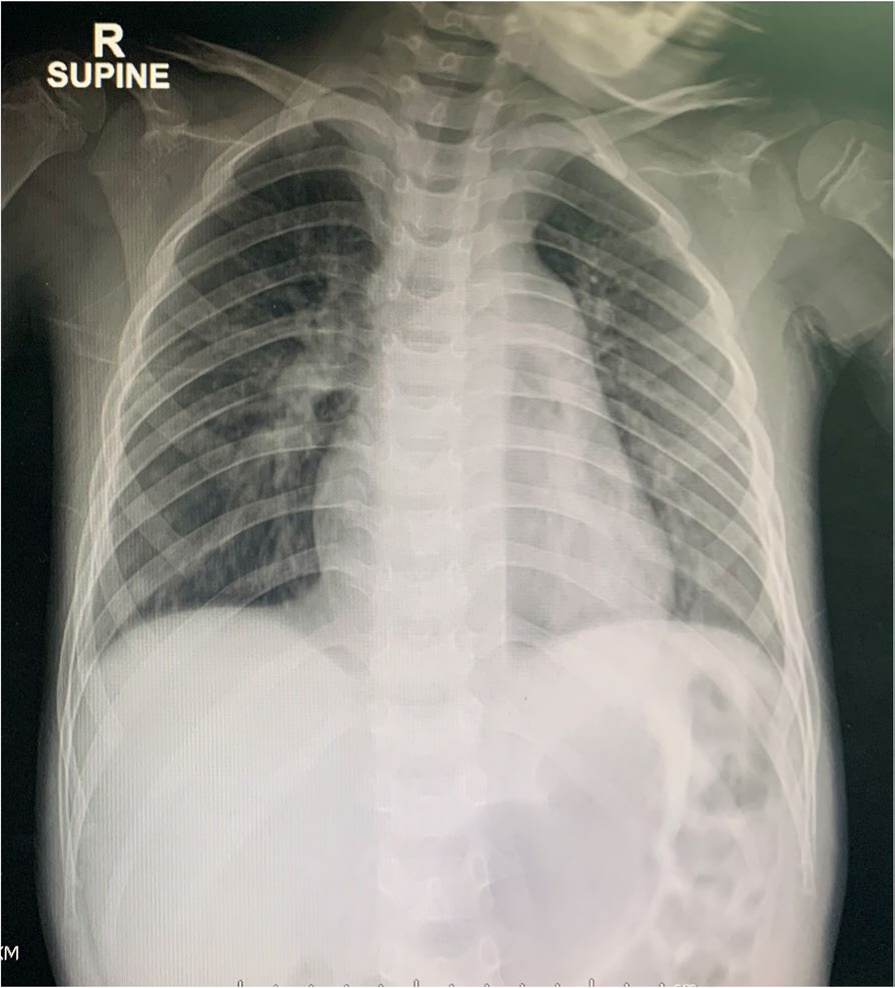
Chest film revealed mild cardiomegaly and prominent pulmonary artery trunk

**Fig. 6:**
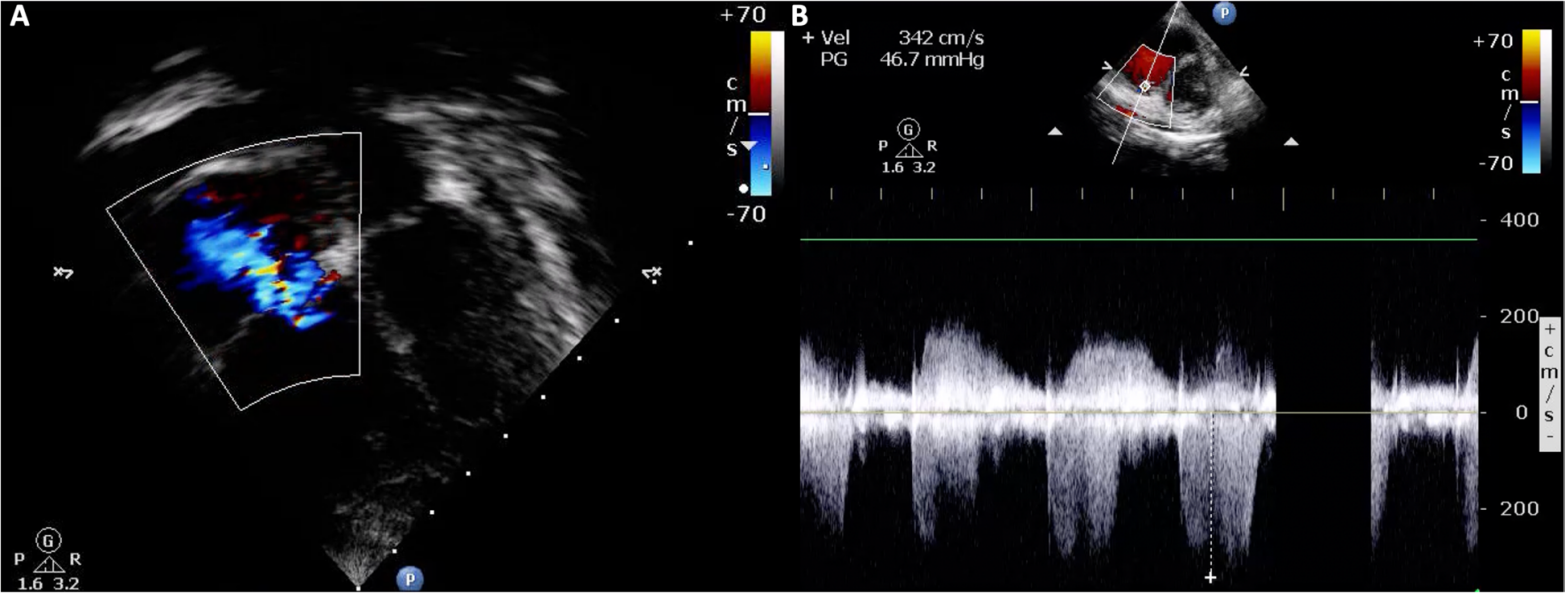
Echocardiographic illustration of Case 2 showed right atrial and ventricular enlargement with moderate TR (**A**), peak velocity 3.4 m/s (**B**)

A 6-year-old boy with autistic spectrum disorder presented with acute dyspnea for 1 day. He had progressive dyspnea and low-grade fever for 1 month and also refused to walk due to his swollen, painful left knee. Physical examination revealed mild tachypnea, tachycardia, oxygen saturation of 90%, mild pale conjunctiva, and diffuse petechiae. Cardiac auscultation indicated a loud S2. His left knee was swollen and tender with limited range of motion. Corkscrew hair covered all parts of his body. His radiographic findings of both knees illustrated metaphyseal fraying and hypolucent lesions at metaphyses of both femurs (Fig. 4). Chest film revealed mild cardiomegaly with prominent pulmonary artery trunk (Fig. 5). Echocardiography showed RA, RV, and main pulmonary artery enlargement, and moderate TR with peak velocity of 3.4 m/s (estimated systolic PAP of 56 mmHg) (Fig. 6). Pulmonary CTA showed a small area of nonspecific ground glass opacity without pulmonary embolism, lung disease, or abnormal vessels. Milrinone and sildenafil were initiated. Right cardiac catheterization measured the PAP of 66/38 (mean = 50) mmHg and derived calculated PVRi was 5.7 WU.m^2^.

Due to the radiographic finding compatibility with scurvy, the exploration of dietary intake revealed mainly rice and egg with few milk, vegetables, and fruit intakes. The vitamin C level was reported as undetectable. Low percentage of transferrin saturation was also detected. Both nutrient supplements were given.

His symptoms improved gradually after 2 weeks of a daily dose of 300 mg of vitamin C, and he could walk within 1 month. The dose of sildenafil was gradually decreased and the medication was discontinued after 2 months with normal echocardiogram indicating normal PAP. His 2-year echocardiographic follow-up illustrated normal cardiac findings without signs of PH.

## Discussion

Pediatric PH shares many common features with PH in adults, but important differences have been recognized. One major characteristic of pediatric PH is its potential reversibility, particularly during infancy, as the pulmonary arterioles undergo developmental transformations to enhance vessel compliance and reduce pulmonary vascular resistance. However, the incidence of PH, particularly pulmonary arterial hypertension (PAH), in childhood is rare and is associated with a poor prognosis, especially when presenting symptoms are related to low CO and heart failure (HF). The diagnosis of PH is based on a mean PAP > 20 mmHg in childhood age older than 3 months [2]. Upon pediatric PH diagnosis, an algorithmic approach is employed to identify underlying diseases to ensure proper classification and treatment [2].

In our report, the first patient presented with a life-threatening episode of low CO, while the second patient complained of dyspnea, a cardinal symptom of HF. Extensive investigations were conducted to uncover the underlying condition in both cases. The common PAH-related conditions such as congenital heart diseases, connective tissue diseases, and lung diseases were not identified. Interestingly, both cases exhibited clinical signs of scurvy, prompting the measurement of ascorbic acid levels, which were found to be undetectable, along with iron deficiency anemia. Remarkably, scurvy is not a common disease in our daily practice nor well-documented as a cause of pediatric PH, except for sporadic case reports. The majority of these reported cases had restrictive dietary habits, particularly in patients with autism spectrum disorder, and all had undetectable vitamin C levels. Notably, abdominal pain was observed only in our first case and a case report from Zavaleta et al. [3] While our second case exhibited common features of leg pain and walking disability, which are known presenting symptoms of scurvy. Therefore, we believe that our series of two cases would provide an additional dimension to the relationship between vitamin C deficiency and pediatric PH, which can be classified as PH with an unclear and/or multifactorial mechanism. Furthermore, both of our cases had a combination of vitamin C and iron deficiency, which may have contributed to the severity of their symptoms [4].

In previous case reports of scurvy-associated PH, the life-threatening episode of PHC related to anesthetic procedures was described. Dean et al. reported cardiac arrest as the presenting symptom [5], while Zalvaleta et al. and Ichiyanagi et al. indicated cases of hemodynamic instability related to anesthesia [3, 6]. In our first case, the patient experienced a sudden cardiovascular collapse in PICU with an estimated systolic PAP of 67 mmHg as documented. Therefore, it is important to be aware that PHC could be a presenting symptom of PH related to vitamin C deficiency in children. We highly recommend measuring Vitamin C and iron levels in pulmonary hypertension children particularly the one who restrictive eating behavior or risk of nutritional deficiencies.

Vitamin C serves as a cofactor for most enzyme reactions, acting as an electron donor and a reducing agent [7]. While vitamin C deficiency can be recognized in some children with developmental problems or restrictive eating habits [8, 9]. Previous case reports, along with theoretical explanations, have highlighted the relationship between vitamin C and pulmonary vasculature from 2 perspectives. Firstly, vitamin C is essential for the hydroxylation of proline residues in Hypoxic Inducible Factor-I alpha (HIF-1 alpha), a major oxygen-sensing transcription factor. HIF-1 alpha is upregulated in hypoxic conditions and correlates to the development of hypoxia induced-pulmonary hypertension [10–12]. Iron also modulates HIF pathway; therefore, combined vitamin C and iron deficiency can increase the severity of pulmonary vasoconstriction [4]. Secondly, vitamin C enhances endothelial nitric oxide (NO) production. NO is one of the potent endogenous pulmonary vasodilators. Therefore, depletion of vitamin C undermines endothelial NO production, leading to less counteraction against pulmonary vasoconstriction [1, 4, 13, 14].

In the majority of the pediatric PH related to vitamin C deficiency, the PH was transient or reversible with supplement ascorbic acid. PAH-targeted therapy was prescribed in 4 cases without significant clinical response [5, 6, 15, 16], whereas most patients demonstrated significant improvement after vitamin C replacement. All cases, including our 2 cases, recovered within 18 months.

## Conclusion

Pediatric PH related to vitamin C deficiency can manifest with a wide range of symptoms, varying from mild and nonspecific to severe life-threatening episodes characterized by PHC. PH associated with scurvy is completely reversible with appropriate investigation, diagnosis, and treatment. Our report aims to highlight the importance of considering nutritional deficiencies as potential confounding factors in pediatric PH, thereby emphasizing the need for comprehensive evaluation and management of these patients.

## Data Availability

Study data can be obtained by sending a request to the corresponding author. Alisa Limsuwan, MD email: alimsuwan@yahoo.com.
